# Supplementary material for: Modulation of large rhythmic depolarizations in human large basket cells by norepinephrine and acetylcholine
Source: Commun Biol. 2024 Jul 20;7:885. doi: 10.1038/s42003-024-06546-2 (PMC11271271; doi:10.1038/s42003-024-06546-2)
Supplement: Supplementary file 2 — Supplementary information [file 42003_2024_6546_MOESM2_ESM.pdf]

Modulation of Large Rhythmic Depolarizations in Human Large Basket Cells by Norepinephrine and Acetylcholine

Supplementary figures and tables

Danqing Yang[1], Guanxiao Qi[1], Jonas Ort[2,7,8], Victoria Witzig[3], Aniella Bak[4], Daniel Delev[2,7,8], Henner Koch[4] and Dirk Feldmeyer [1,5,6 #]

1 Research Center Juelich, Institute of Neuroscience and Medicine 10, Research Center Juelich, 52425 Juelich, Germany.
2 Department of Neurosurgery, Faculty of Medicine, RWTH Aachen University Hospital, Aachen, Germany
3 Department of Neurology, RWTH Aachen University Hospital, 52074 Aachen, Germany.
4 Department of Neurology, Section Epileptology, RWTH Aachen University Hospital, 52074 Aachen, Germany.
5 Department of Psychiatry, Psychotherapy, and Psychosomatics, RWTH Aachen University Hospital, 52074 Aachen, Germany.
6 Jülich-Aachen Research Alliance, Translational Brain Medicine (JARA Brain), Aachen, Germany.
7 Neurosurgical Artificial Intelligence Laboratory Aachen (NAILA), RWTH Aachen University Hospital, 52074 Aachen, Germany
8 Center for Integrated Oncology, Universities Aachen, Bonn, Cologne, Düsseldorf (CIO ABCD), Germany.

# Correspondence should be addressed to Dirk Feldmeyer at d.feldmeyer@fz-juelich.de or dfeldmeyer@ukaachen.de

Tab. S1 Patient demographic and clinical data for the tissue used in this study.

Patient Number	Patient Age	Diagnosis	Gender	Brain region	Detection of Seizures	Distance to lesion (mm)	Growth type	Intracellular recordings		
								Cortical layer	Number of neurons recorded	LRD+ neurons
**1**	35	Cavernoma	Female	Temporal cortex left	Yes	21.4	Non infiltrative	L2/3	2 PCs<br>3 INs	2 INs
**2**	40	Tumor	Female	Frontal cortex right	No	20	Non infiltrative	L2/3	2 INs	-
**3**	50	Tumor	Female	Occipital cortex right	No	12.6	Infiltrative	L2/3	2 PCs<br>2 INs	-
**4**	75	Tumor	Female	Frontal cortex right	Yes	35	Infiltrative	L2/3	3 PCs<br>2 INs	-
**5**	56	Tumor	Female	Frontal cortex right	Yes	31.5	Infiltrative	L2/3	1 PC<br>1 IN	-
**6**	47	Tumor	Male	Frontal cortex right	Yes	17.2	Infiltrative	L2/3	3 PCs<br>1 IN	-
**7**	24	Epilepsy	Female	Temporal cortex right	Yes	25.1	Non infiltrative	L2/3	6 PCs<br>1 IN	2 PCs
**8**	62	Cavernoma	Female	Temporal cortex right	Yes	13.4	Non infiltrative	L2/3	5 PCs<br>1 IN	-
**9**	55	Tumor	Male	Frontal cortex left	Yes	17.5	Infiltrative	L2/3	2 PCs<br>2 INs	-
**10**	71	Tumor	Female	Precental cortex right	Yes	27.5	Non infiltrative	L2/3	2 PCs<br>4 INs	1 IN
**11**	16	Tumor & Epilepsy	Male	Temporal cortex right	Yes	35.5	Non infiltrative	L2/3	2 PCs<br>3 INs	2 INs
**12**	66	Tumor	Female	Temporal cortex right	No	13	Infiltrative	L2/3	3 PCs<br>2 INs	2 INs
**13**	8	Unknown	Male	Temporal cortex right	Yes	33.8	Non infiltrative	L2/3	4 PCs<br>2 INs	-
**14**	64	Tumor	Male	Parietal cortex left	Yes	15.2	Infiltrative	L2/3	1 PC<br>2 INs	-
**15**	40	Epilepsy	Female	Temporal cortex	Yes	40	Non infiltrative	L2/3	3 PCs<br>4 INs	3 INs
**16**	42	Tumor	Female	Temporal cortex	Yes	23	Infiltrative	L2/3	6 PCs<br>3 INs	1 IN
**17**	65	Tumor	Male	Frontal cortex right	Yes	24.2	Infiltrative	L2/3	3 PCs<br>4 INs	2 INs
**18**	44	Tumor	Male	Frontal cortex left	Yes	26.6	Infiltrative	L2/3	1 PC<br>3 INs	1 IN
**19**	50	Tumor	Male	Temporal cortex right	No	20.4	Non infiltrative	L2/3	1 PC<br>4 INs	-
**20**	45	Epilepsy	Male	Temporal cortex left	Yes	40	Non infiltrative	L2/3	10 PCs<br>3 INs	-
**21**	37	Epilepsy	Female	Temporal cortex left	Yes	25	Non infiltrative	L2/3	4 PCs<br>2 INs	3 PCs
								L5/6	4 PCs<br>1 IN	1IN
**22**	54	Tumor	Male	Temporal cortex right	Yes	30	Infiltrative	L2/3	11 PCs<br>2 INs	-
**23**	37	Tumor	Male	Frontal cortex right	Yes	10	Infiltrative	L2/3	3 PCs<br>7 INs	2 INs
**24**	32	Tumor	Female	Temporal cortex left	Yes	15.8	Infiltrative	L2/3	4 PCs<br>4 INs	2 PCs<br>1 IN

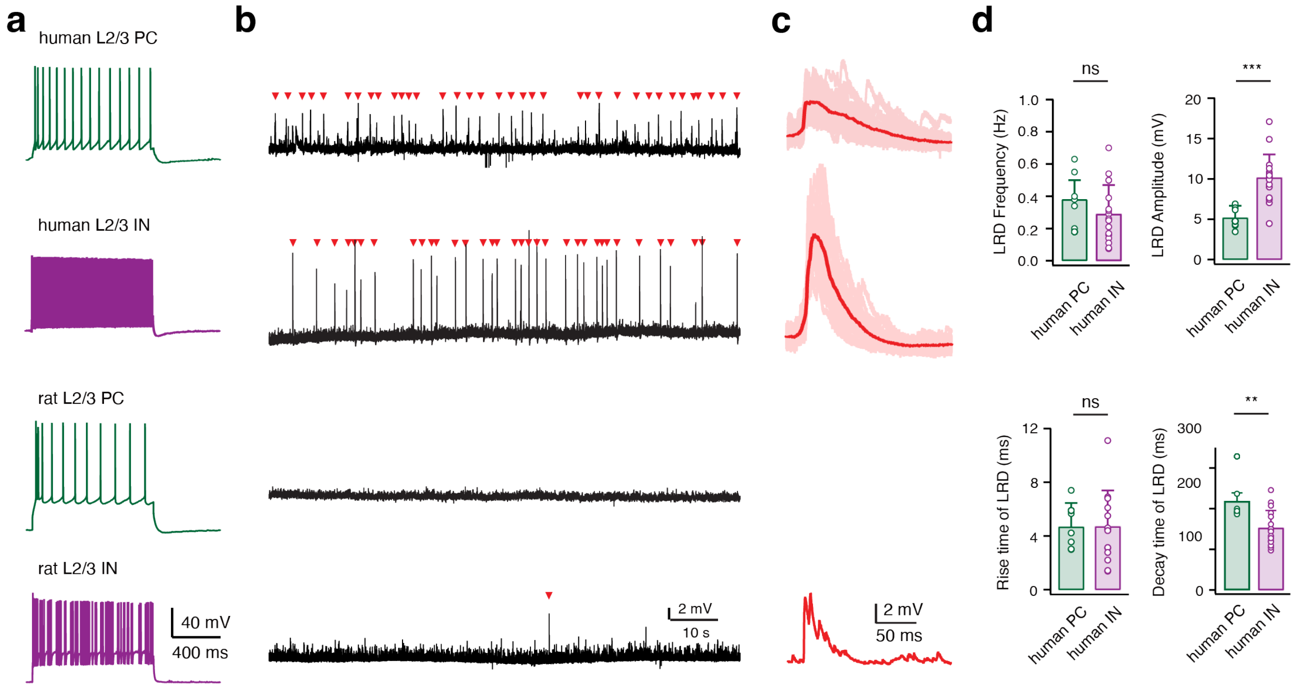

Fig. S1 Regular network events were not observed in L2/3 neurons of adult rat prefrontal or temporal cortex.

a Representative firing patterns of a human L2/3 PC, a human L2/3 interneuron, a rat L2/3 PC and a rat L2/3 interneuron. Firing patterns of PCs are shown in green while those of interneurons are shown in purple.

b Corresponding current-clamp recording traces are obtained from the same neuron in a. Spontaneously occurring LRDs are marked in red.

c The average and individual LRDs are superimposed and given in a darker and lighter red, respectively.

d Histograms comparing the frequency, amplitude, rise time and decay time of LRDs between PCs (n = 7) and interneurons (n = 17). ** $P < 0.01$, *** $P < 0.001$ for the Wilcoxon Mann–Whitney U test.

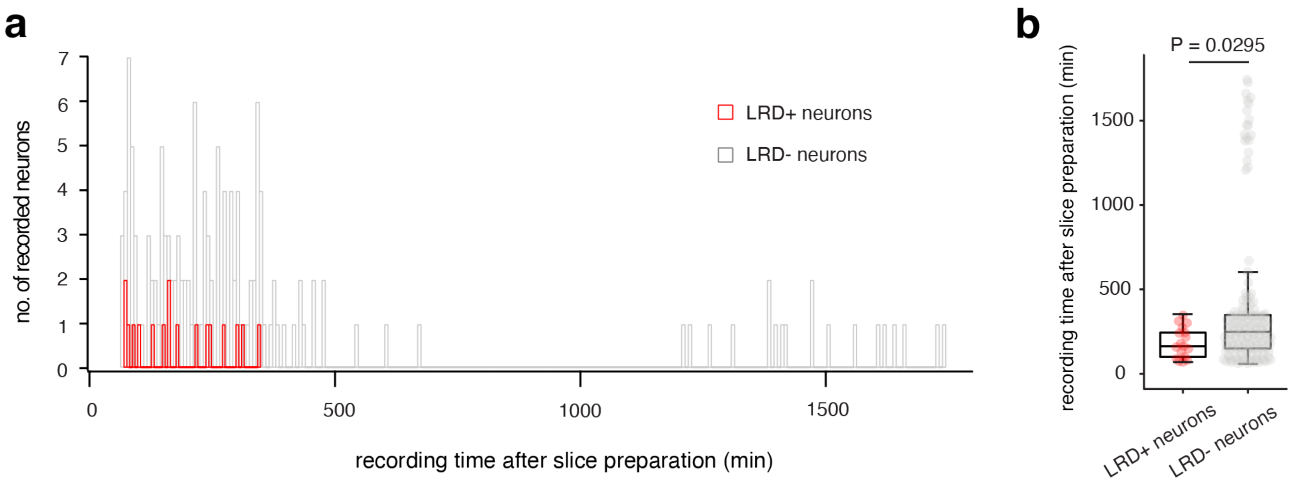

Fig. S2 LRDs were observed only within 6 hours (360 min) after slice preparation

a Recording time after slice preparation of LRD-positive and LRD-negative neurons in human L2/3. Histograms were constructed with 6 min bins. LRD+ neurons are shown in red while LRD- neurons are shown in gray.

b Box plot comparing the recording time after slice preparation for LRD+ (n = 17) and LRD- (n = 146) neurons. P =0.0295 for the Wilcoxon Mann–Whitney U test.

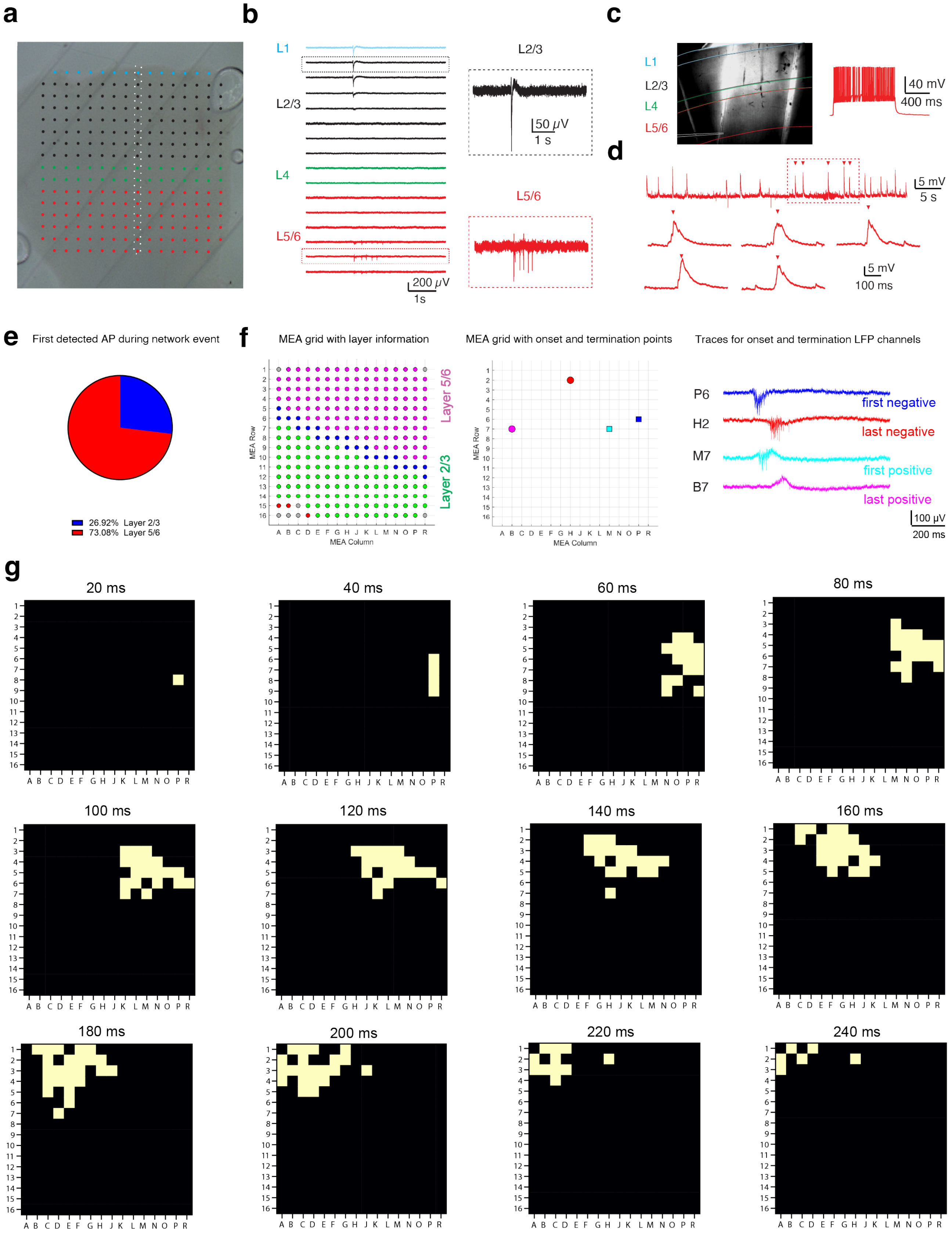
a
b
c
d
e
f
g
L1
L2/3
L4
L5/6
200 µV
1s
50 µV
1 s
40 mV
400 ms
5 mV
5 s
5 mV
100 ms
First detected AP during network event
26.92% Layer 2/3
73.08% Layer 5/6
MEA grid with layer information
Layer 5/6
Layer 2/3
MEA Row
MEA Column
MEA grid with onset and termination points
Traces for onset and termination LFP channels
P6
first negative
H2
last negative
M7
first positive
B7
last positive
100 µV
200 ms
20 ms
40 ms
60 ms
80 ms
100 ms
120 ms
140 ms
160 ms
180 ms
200 ms
220 ms
240 ms

Fig. S3 Synchronous network events were observed in human cortical layer 6.

a A typical human slice culture is shown with all cortical layers (L1–L6) and overlay of the MEA on the slice. The MEA channels in L1, L2/3, L4 and L5/6 are marked by light blue, black, green and red dots, respectively.

b Representative voltage traces from 16 MEA channels. Black and red insets show a detailed view of LFPs in L2/3 and L5/6, respectively.

c Position of the recorded layer 6 interneuron in a human acute slice. Layer borders are indicated by the same color code as in (b). Firing pattern of the recorded neuron is shown on the right.

d Whole cell intracellular recordings from a L6 interneuron in human neocortex showing LRD activity. 5 marked consecutive LRDs are shown at higher magnification at the bottom.

e Pie chart shows that most detected Network activities are initiated in Layer 5/6.

f Representation of the Layer in relation to the MEA grid and onset and termination point of negative and positive LFPs during the network event (on the right: the traces of the first and last LFP detected for one network event).

g Time course of detected negative LFPs on the MEA grid in 20 ms bins, note the complex propagation pattern with vertical and horizontal propagation.

Tab. S2 Morphological and electrophysiological properties of LRD+ and LRD- human cortical L2/3 interneurons.

Italic bold font indicates significant differences; *P < 0.05, **P < 0.01, ***P < 0.001 for Wilcoxon Mann-Whitney U test.

	LRD+ L2/3 INs	LRD- L2/3 INs	Mann-Whitney Test
	Morphological properties (n = 8 for each group)		
somatic area (μm^2)	170.2 ± 44.1	186.2 ± 84.1	0.6454
dendritic length (µm)	4171.2 ± 1210.4	2080.1 ± 1105.0	****0.0207***
axonal length (µm)	40778.6 ± 8197.8	12485.7 ± 5402.8	******0.0003***
no. of dendrites	6.0 ± 1.6	3.9 ± 1.2	0.3915
H-fieldspan of dendrite (µm)	546.2 ± 176.1	212.1 ± 104.0	******0.0003***
V-fieldspan of dendrite (µm)	652.4 ± 193.8	308.3 ± 216.8	*****0.0047***
H-fieldspan of axon(µm)	1227.6 ± 427.3	604.0 ± 405.0	****0.0140***
V-fieldspan of axon (µm)	1007.5 ± 284.1	859.3 ± 404.7	0.4634
	Electrophysiological properties (n = 13 for each group)		
resting membrane potential (mV)	-67.0 ± 6.4	-66.0 ± 6.8	0.7399
Input resistance (MΩ)	192.4 ± 85.1	298.5 ± 117.9	****0.0278***
AP half- width (ms)	0.48 ± 0.21	0.37 ± 0.12	0.2406
AP amplitude (mV)	79.2 ± 9.5	84.4 ± 7.5	0.3338
AP threshold (mV)	-41.2 ± 2.9	-42.3 ± 3.2	0.3760
AHP amplitude (mV)	23.7 ± 5.1	25.0 ± 3.7	0.7689
AP latency (ms)	196.8 ± 155.5	143.6 ± 101.1	0.7283
frequency- current slope (Hz/100 pA)	30.2 ± 8.7	40.7 ± 17.6	0.1164
Adaptation (ISI_2/ISI_{10})	0.75 ± 0.24	0.79 ± 0.24	0.6981

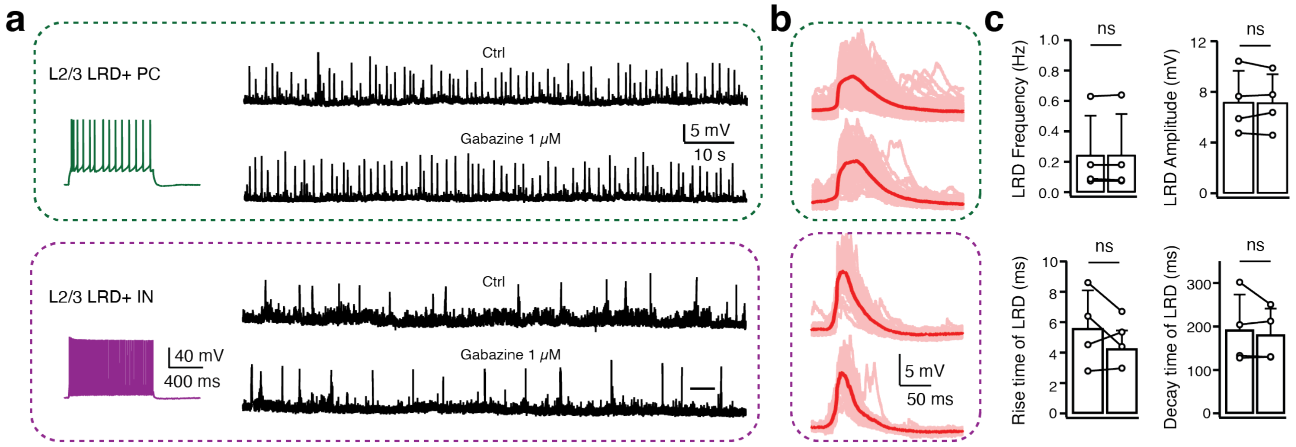

Fig. S4 Gabazine has no effect on LRD properties.

a Left, representative firing patterns of a LRD-positive human L2/3 PC (top) and a interneuron (bottom). Firing patterns of PCs are shown in green while those of interneurons are shown in purple. Corresponding current-clamp recording traces are obtained from the same neuron shown on the left.

b The average and individual LRDs are superimposed and given in a darker and lighter red, respectively.

c Histograms comparing the frequency, amplitude, rise time and decay time of LRDs under control and gabazine conditions (n = 4). Ns, not significant for the Wilcoxon Mann–Whitney U test.

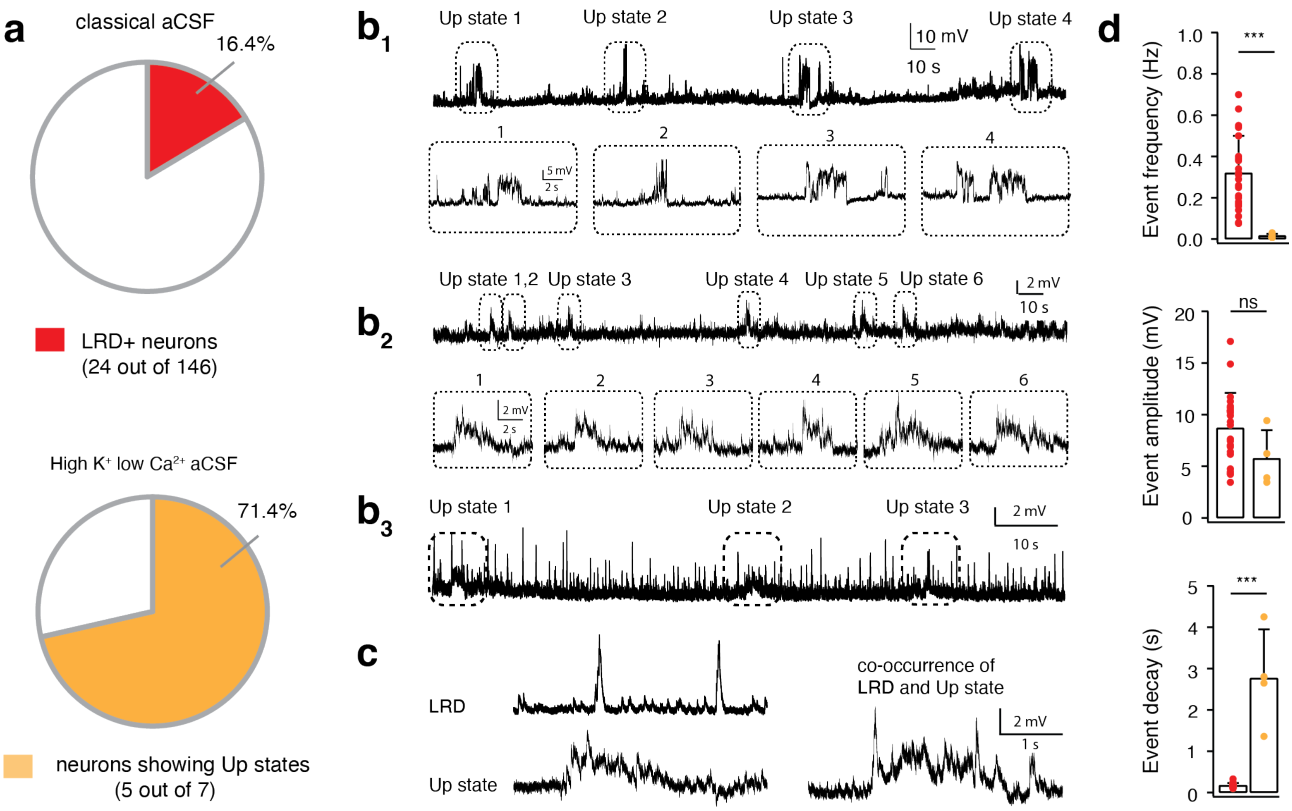

Fig. S5 Comparison of high K⁺/low Ca²⁺ ACSF-induced 'Up' states with LRDs.

a Top, pie chart illustrating the percentage of recorded LRD-positive neurons when slices were perfused with classical aCSF containing 2.5 mM K^+ and 2 mM Ca^{2+}. Bottom, pie chart showing the percentage of neurons displaying slow oscillations when slices were perfused with aCSF containing 3.5 mM K^+ and 1 mM Ca^{2+}.

b Neurons showing typical 'Up' states containing both depolarizing and hyperpolarizing components when perfused with high K^+/low Ca^{2+} aCSF. **b$_1$** Current clamp recording trace of a human L2/3 interneuron, individual Up state is marked and enlarged at the bottom. **b$_2$** Current clamp recording trace of a human L2/3 PC, individual Up state is marked and enlarged at the bottom. **b$_3$**, Current clamp recording of a human L2/3 PC showing co-occurrence of LRDs and Up states.

c Left, comparison of LRD and Up states at the same time scale. Right, co-occurrence of LRD during an 'Up' state.

d Histograms comparing the frequency, amplitude and decay time of LRDs (n = 24) and Up states (n = 4). LRDs are indicted by red dots while Up states in orange. *** P < 0.001 for the Wilcoxon Mann–Whitney U test; ns, not significant.

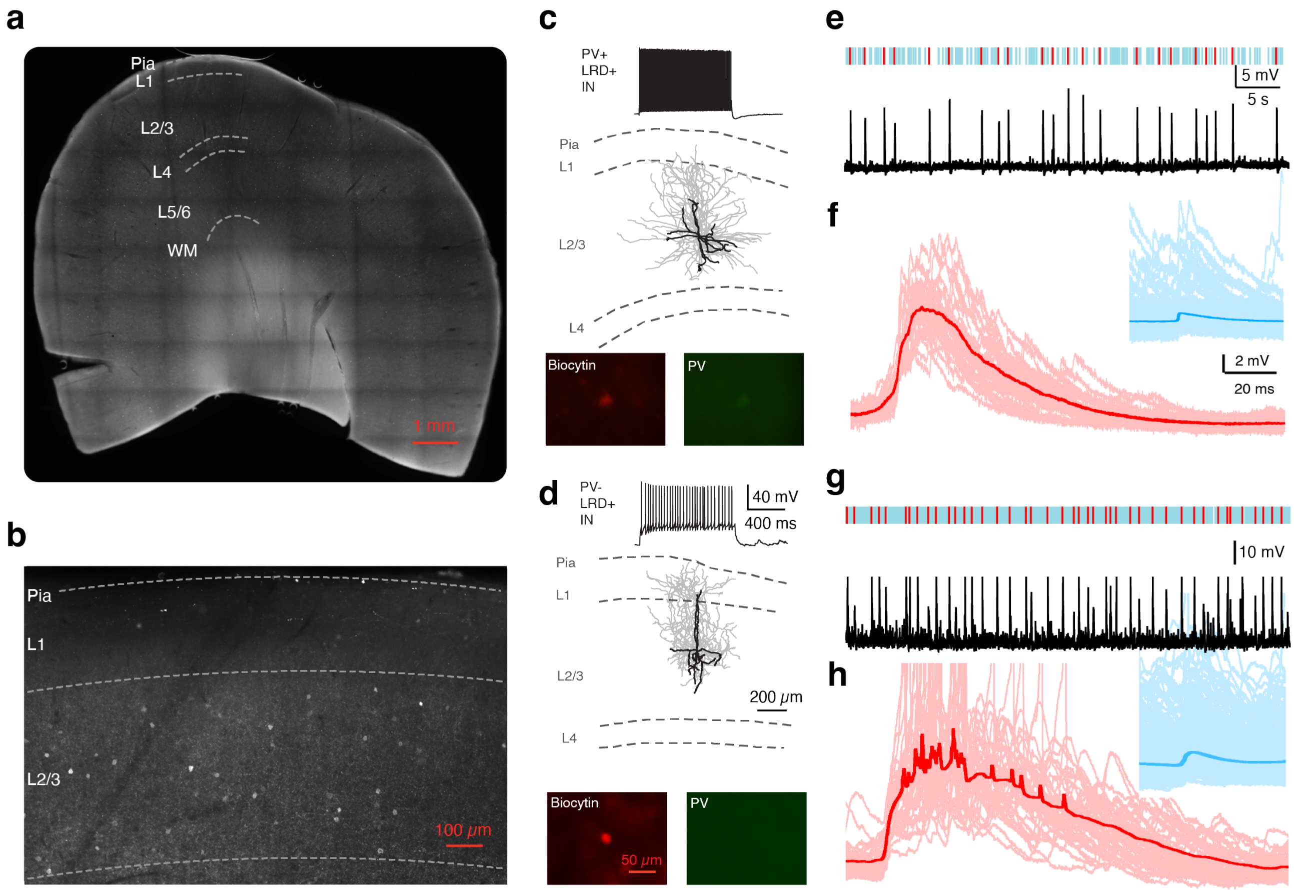

Fig. S6 Large basket cells showing LRDs in L2/3 human cortex are either parvalbumin (PV) positive or negative.

a Representative example of a human brain slice after antibody labelling for PV. Layer borders are indicated by dashed white lines.

b PV positive interneurons are more abundant in layer 2/3 than in layer 1. Layer borders are indicated with dashed white lines.

c, d Top: Representative firing patterns of a PV+, LRD+ human L2/3 interneuron (c) and a PV- LRD+ human L2/3 interneuron (d). Middle: Corresponding morphological reconstructions of the same neurons shown above. The somatodendritic domain is given in black and the axons in gray. Bottom: Neurons were recorded using whole-cell patch-clamp technique and simultaneously filled with biocytin and the fluorescent dye Alexa 594 (red). Antibody labeling was performed to test for the expression of PV (green).

e, g A 50 s recording was obtained from the same PV+ (e) and PV- interneuron (g), respectively. Normal EPSPs are marked in blue while LRDs are marked in red.

f, h Left: The average and individual LRDs are superimposed and given in a darker and lighter red, respectively. Right: The average and individual EPSPs are superimposed and given in a darker and lighter blue, respectively. Events were extracted and analyzed from the continuous recordings shown in **e** and **g**.

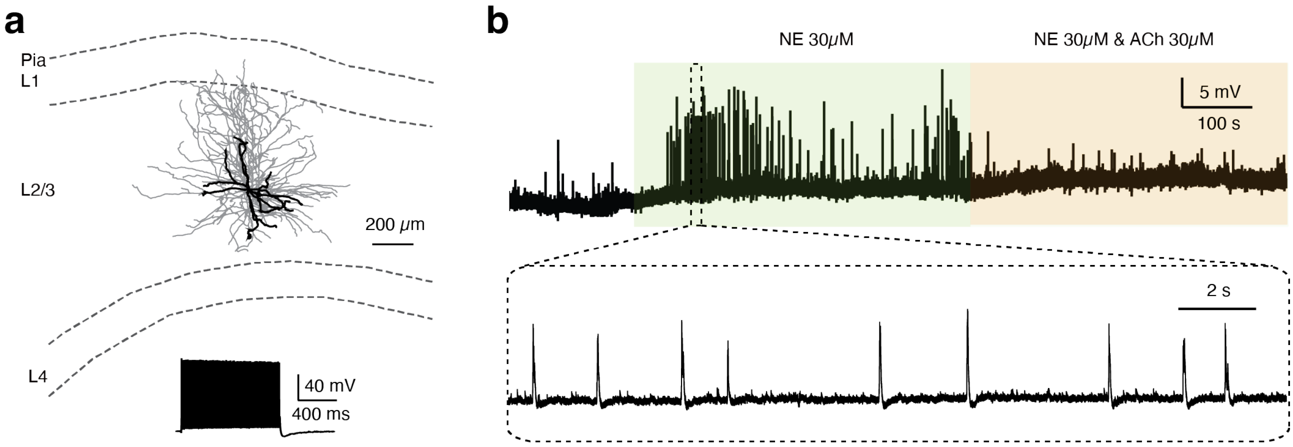

Fig. S7 ACh suppresses the NE-induced LRDs in L2/3 human interneurons

a Top: Representative morphological reconstruction of a LRD+ human L2/3 interneuron. Bottom: Corresponding firing pattern of the same neuron shown above.

b Representative current-clamp recordings with bath application of 30 μM NE causing an increase in LRD frequency in the same neuron shown in a. The NE effect is reversed by 30 μM ACh.

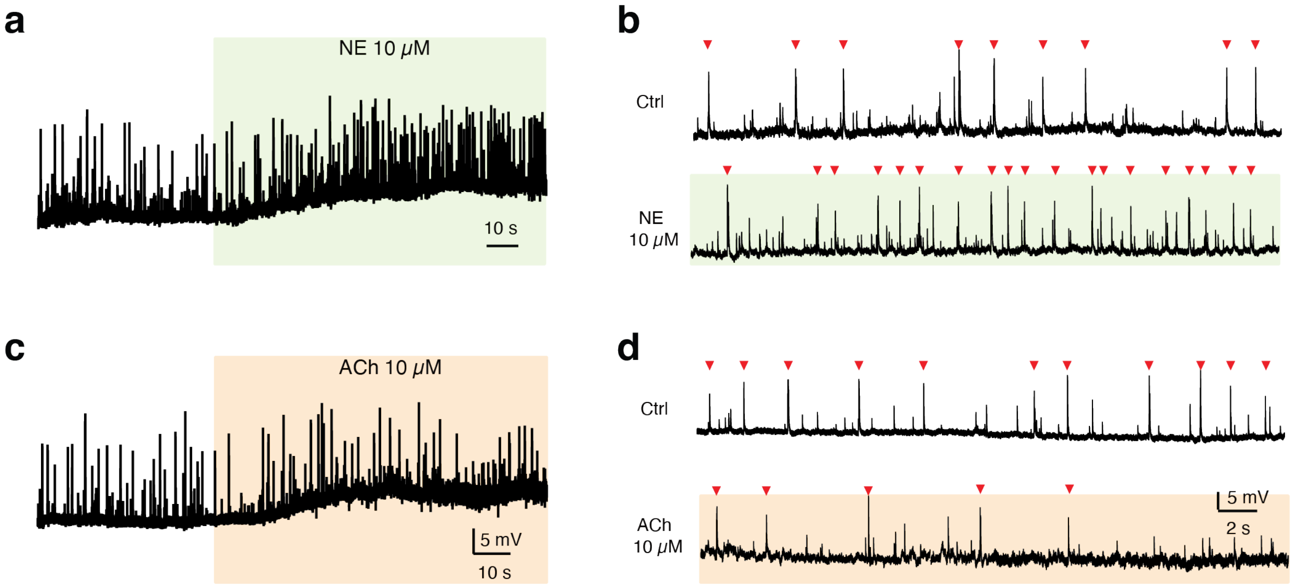

Fig. S8 Low concentrations of ACh (10 μM) and NE (10 μM) show similar modulation of LRDs in L2/3 human interneurons

a Representative current-clamp recordings with bath application of 10 μM NE showing an increase of LRD frequency and a membrane potential depolarization in a human L2/3 interneuron.

b Enlarged recording traces under control and 10 μM NE conditions. LRDs are marked by red arrowheads.

c Representative current-clamp recordings with bath application of 10 μM ACh showing a decrease of LRD frequency and a membrane potential depolarization in a human L2/3 interneuron.

d Enlarged recording traces under control and 10 μM ACh conditions. LRDs are marked by red arrowheads.

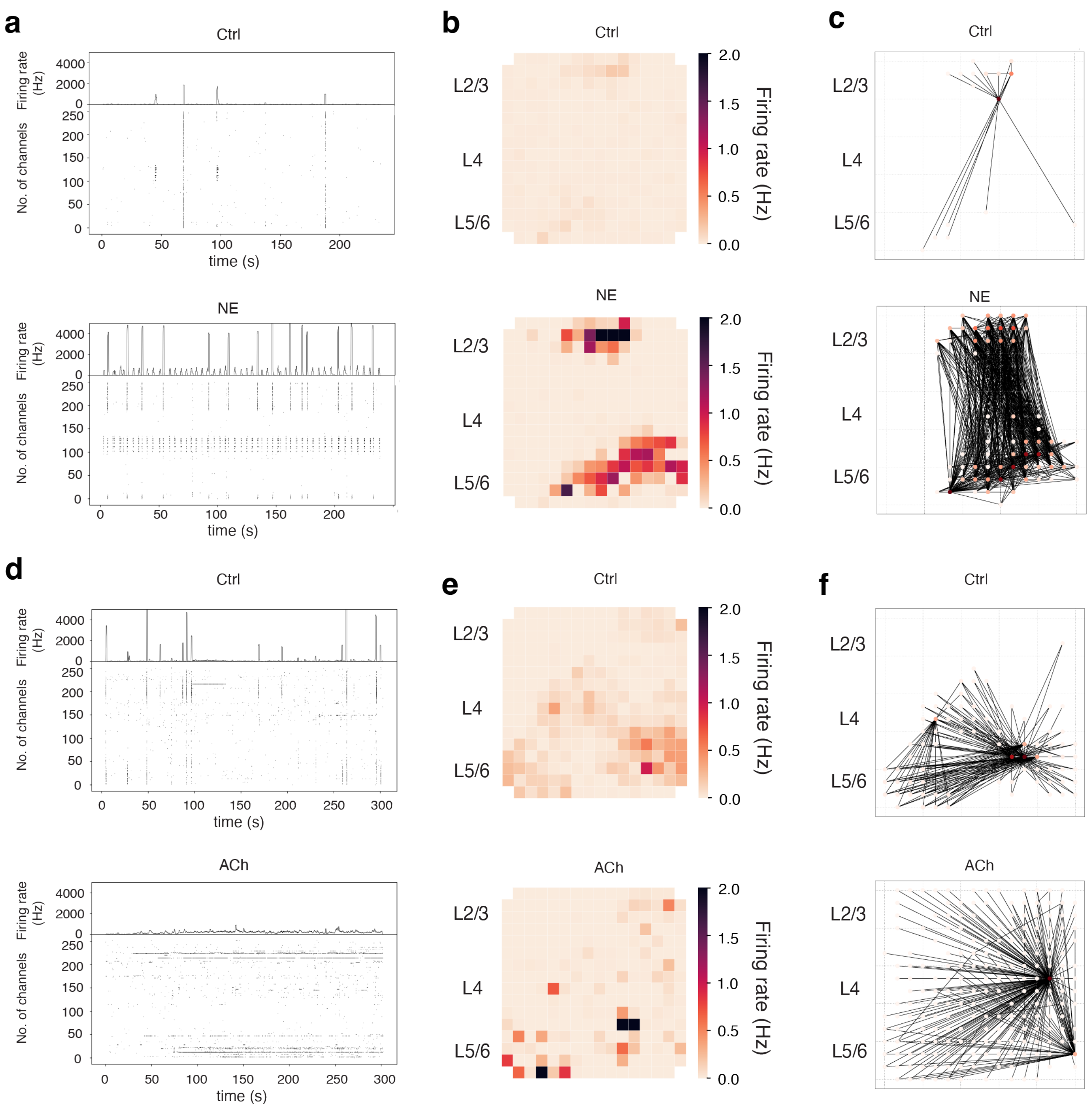

Fig. S9. NE causes synchronization while ACh promotes desynchronization of neuronal firing.

a Raster plots of the detected action potentials over a 5 min recording period under control condition and in the presence of NE showing an increase in synchronous firing.

b Heatmap of the average firing rate over the MEA grid from a 5 min recording in control condition and in the presence of NE showing an increase synchronous firing in L2/3 and deep layers.

c Graph analysis of MEA recordings over a 5 min period under control condition and in the presence of NE showing an increase of degree of centrality. A line connecting two MEA channels (*nodes of the graph*) represents an *edge of the graph*. Two *nodes* are connected by one *edge* only if they spike synchronously within a bin of 200 ms. Degree centrality is calculated from number of connected *nodes* and number of *edges* used for these connections.

d Raster plots of the detected action potentials over the 5 min recording period under control condition and in the presence of ACh showing a decrease in synchronous firing.

e Heatmap of the average firing rate over the MEA grid from a 5 min recording in control condition and in the presence of ACh showing a decrease in temporal and spatial correlation of global AP firing.

f Graph analysis of MEA recordings over a 5 min period under control condition and in the presence of ACh showing a decrease of degree of centrality. For details s. c).

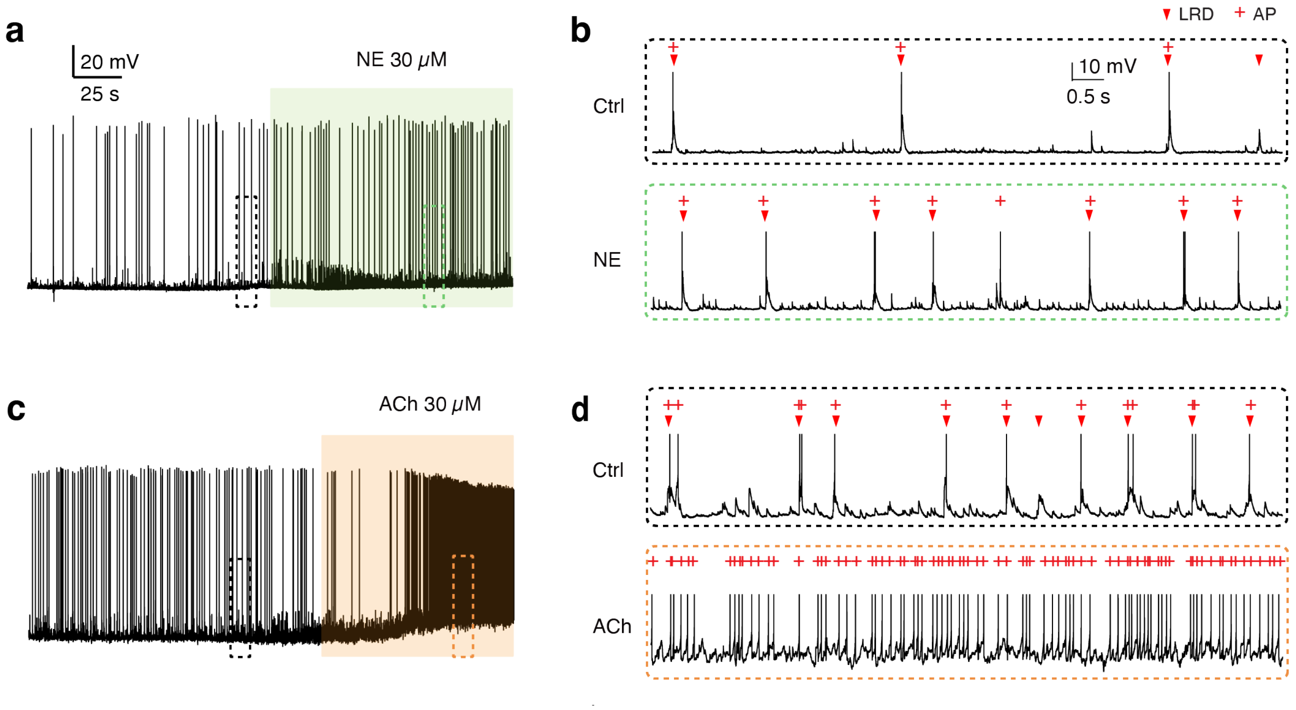

Fig. S10. NE and ACh increase AP firing rates via different mechanisms

a Representative current-clamp recordings with bath application of 30 µM NE showing an increase of LRD frequency and AP firing rates in a human L2/3 interneuron.

b Enlarged recording traces under control and 30 µM NE conditions. LRDs and APs are marked by red arrowheads and red plus mark, respectively.

c Representative current-clamp recordings with bath application of 30 µM ACh showing a strong membrane potential depolarization and increase of AP firing rates in a human L2/3 interneuron.

d Enlarged recording traces under control and 30 µM ACh conditions. LRDs and APs are marked by red arrowheads and red plus mark, respectively.
