# Supplementary material for: Modulation of large rhythmic depolarizations in human large basket cells by norepinephrine and acetylcholine
Source: Commun Biol. 2024 Jul 20;7:885. doi: 10.1038/s42003-024-06546-2 (PMC11271271; doi:10.1038/s42003-024-06546-2)
Supplement: Supplementary file 3 — Description of Additional Supplementary Files [file 42003_2024_6546_MOESM3_ESM.pdf]

Description of Additional Supplementary Files

File name: Supplementary Data 1
Description: The source data behind the Fig.1 in the paper

File name: Supplementary Data 2
Description: The source data behind the Fig.2 in the paper

File name: Supplementary Data 3
Description: The source data behind the Fig.3 in the paper

File name: Supplementary Data 4
Description: The source data behind the Fig.4 in the paper
