# Supplementary material for: Modulation of large rhythmic depolarizations in human large basket cells by norepinephrine and acetylcholine
Source: Commun Biol. 2024 Jul 20;7:885. doi: 10.1038/s42003-024-06546-2 (PMC11271271; doi:10.1038/s42003-024-06546-2)
Supplement: Supplementary file 8 — Reporting Summary [file 42003_2024_6546_MOESM8_ESM.pdf]

nature portfolio

Corresponding author(s): Dirk Feldmeyer

Last updated by author(s): Jun 18, 2024

Reporting Summary

Nature Portfolio wishes to improve the reproducibility of the work that we publish. This form provides structure for consistency and transparency in reporting. For further information on Nature Portfolio policies, see our Editorial Policies and the Editorial Policy Checklist.

Statistics

For all statistical analyses, confirm that the following items are present in the figure legend, table legend, main text, or Methods section.

n/a	Confirmed	
☐	☒	The exact sample size (*n*) for each experimental group/condition, given as a discrete number and unit of measurement
☐	☒	A statement on whether measurements were taken from distinct samples or whether the same sample was measured repeatedly
☐	☒	The statistical test(s) used AND whether they are one- or two-sided<br>*Only common tests should be described solely by name; describe more complex techniques in the Methods section.*
☐	☒	A description of all covariates tested
☐	☒	A description of any assumptions or corrections, such as tests of normality and adjustment for multiple comparisons
☐	☒	A full description of the statistical parameters including central tendency (e.g. means) or other basic estimates (e.g. regression coefficient) AND variation (e.g. standard deviation) or associated estimates of uncertainty (e.g. confidence intervals)
☐	☒	For null hypothesis testing, the test statistic (e.g. *F*, *t*, *r*) with confidence intervals, effect sizes, degrees of freedom and *P* value noted<br>*Give P values as exact values whenever suitable.*
☒	☐	For Bayesian analysis, information on the choice of priors and Markov chain Monte Carlo settings
☒	☐	For hierarchical and complex designs, identification of the appropriate level for tests and full reporting of outcomes
☒	☐	Estimates of effect sizes (e.g. Cohen's *d*, Pearson's *r*), indicating how they were calculated

Our web collection on statistics for biologists contains articles on many of the points above.

Software and code

Policy information about availability of computer code

Data collection	Electrophysiological data collection was done with patchmaster software (HEKA), 3D neuronal morphological reconstruction was done using NEUROLUCIDA software (MBF Bioscience), MEA recordings were performed using the Multi-Channel Experimenter (Multi Channel Systems MCS GmbH).
Data analysis	Analysis of electrophysiological data was done with Igor Pro 6 (WaveMetrics) using custom written macros, analysis of 3D-reconstructed neurons was done with NEUROEXPLORER software (MBF Bioscience), MEA recordings were analyzed using custom-written programs in Python, statistical analysis was done with standard functions in Igor Pro.

For manuscripts utilizing custom algorithms or software that are central to the research but not yet described in published literature, software must be made available to editors and reviewers. We strongly encourage code deposition in a community repository (e.g. GitHub). See the Nature Portfolio guidelines for submitting code & software for further information.

Data

Policy information about availability of data

All manuscripts must include a data availability statement. This statement should provide the following information, where applicable:

- Accession codes, unique identifiers, or web links for publicly available datasets
- A description of any restrictions on data availability
- For clinical datasets or third party data, please ensure that the statement adheres to our policy

All raw and processed data will be made available upon request.

Research involving human participants, their data, or biological material

Policy information about studies with human participants or human data. See also policy information about sex, gender (identity/presentation), and sexual orientation and race, ethnicity and racism.

Reporting on sex and gender	We collected data from 24 patients including 15 females and 9 males.
Reporting on race, ethnicity, or other socially relevant groupings	n/a
Population characteristics	Patients are aging from 8 to 75 years old, all patients underwent neurosurgical resections because of pharmaco-resistant epilepsy or tumor removal. Brain tissue was surgically removed in 11 cases from frontal cortex, 8 cases from temporal cortex, in one case each from the occipital, precental and parietal cortex.
Recruitment	Surgical specimens were obtained from local hospitals (RWTH Aachen Hospital) in collaboration with neurosurgeons. Although bias could be present if there are biases in characteristics of individuals undergoing surgery for epilepsy or tumor, or of the subset of individuals surgery who choose to donor brain tissue, in this study we found neuron properties did not correlate with patient age, gender, neocortical subregion of the tissue or pathophysiological condition.
Ethics oversight	Approval of the Ethics Committee for RWTH Aachen University has been obtained for the study protocol. From all patients, written informed consent to use spare neocortical tissue acquired during the surgical approach was obtained.

Note that full information on the approval of the study protocol must also be provided in the manuscript.

Field-specific reporting

Please select the one below that is the best fit for your research. If you are not sure, read the appropriate sections before making your selection.

☒ Life sciences ☐ Behavioural & social sciences ☐ Ecological, evolutionary & environmental sciences

For a reference copy of the document with all sections, see nature.com/documents/nr-reporting-summary-flat.pdf

Life sciences study design

All studies must disclose on these points even when the disclosure is negative.

Sample size	Sample sizes were determined based on power analysis, with expected variance and effect sizes based on literature and experience
Data exclusions	Data were not cluded from analysis unless not reaching the required noise level or recording quality.
Replication	All experiments in the study were replicated within the study by different experimenters
Randomization	Randomization of experiments with internal controls was done by randomly assigning trial conditions.
Blinding	Blinding was not possible. Experiments were done with internal controls, where as much as possible the experimental conditions were randomly varied.

Reporting for specific materials, systems and methods

We require information from authors about some types of materials, experimental systems and methods used in many studies. Here, indicate whether each material, system or method listed is relevant to your study. If you are not sure if a list item applies to your research, read the appropriate section before selecting a response.

Materials & experimental systems

n/a	Involved in the study
☐	☒ Antibodies
☒	☐ Eukaryotic cell lines
☒	☐ Palaeontology and archaeology
☐	☒ Animals and other organisms
☒	☐ Clinical data
☒	☐ Dual use research of concern
☒	☐ Plants

Methods

n/a	Involved in the study
☒	☐ ChIP-seq
☒	☐ Flow cytometry
☒	☐ MRI-based neuroimaging

Antibodies

Antibodies used	Rabbit-anti-Parvabumin primary antibody (1:120, ab11427, Abcam), Donkey-anti-Rabbit Alexa Fluor secondary antibody (1:400, A21207, Invitrogen)
Validation	The PV antibody in the study have been widely used in the literature, and have been validated in our lab in Ding et al. Layer-Specific Inhibitory Microcircuits of Layer 6 Interneurons in Rat Prefrontal Cortex. Cerebral Cortex. 2021.

Animals and other research organisms

Policy information about studies involving animals; ARRIVE guidelines recommended for reporting animal research, and Sex and Gender in Research

Laboratory animals	Wistar rats (Charles River) aged 40–55 postnatal days
Wild animals	n/a
Reporting on sex	either sex
Field-collected samples	n/a
Ethics oversight	All experimental procedures involving animals were performed in accordance with the guidelines of the Federation of European Laboratory Animal Science Association, the EU Directive 2010/63/EU, and the German animal welfare law.

Note that full information on the approval of the study protocol must also be provided in the manuscript.
